# Annotations

**Published:** 1903-12-19

**Authors:** 


### Annotations.

### St. Bartholomew's Hospital.

When we decided to try and save St. Bartholomew's Hospital from the ruin with which it was threatened by the patchwork scheme formulated by the Mansion House Committee, we were fully alive to the fact that the hope of success was, in general estimation, slight indeed. We have always believed in a policy of thorough, which has once more triumphed, for as the *Pall Mall Gazette* properly points out, the results obtained in the case of Bart's is a decided victory for efficiency. We have also to place on record our acknowledgment of the invaluable support which has been given to the movement by the *Westminster Gazette*, and of the substantial aid rendered by the *Times*, the *Daily Chronicle*, the *Standard*, the *Morning Post*, the *Morning Advertiser*, the *Pall Mall*, the *Financial News*, the *Daily News*, the *Daily Mail*, the *Morning Leader*, and the *Daily Express*. The result proves once more the power for good wielded by the fourth estate, and this power has been immeasurably increased in the present case by the co-operation of the *Lancet* and the *British Medical Journal*, which have loyally supported the policy of efficiency and thoroughness from the outset of our crusade. The acting medical staff of St. Bartholomew's are also to be congratulated upon the course they have taken in the matter. We congratulate the treasurer, the Committee of Almoners, and the House Committee, upon the courage and spirit they have shown in retiring from an untenable position. We hope that all parties will now unite to secure the best possible plan. If this be done, we are confident that within the next five years money will be provided in sufficient quantity, and that St. Bartholomew's will be made worthy of the best traditions of the City of London. The medical staff wisely insist that the acquisition of more ground will necessitate the reconsideration of all previous plans for wards and other new buildings. The Governors must support this view by agreeing to postpone building operations of every kind until there is in existence a perfected plan, complete in all particulars dealing with the whole site available, including the additional land. Economical and financial considerations impose upon the Governors the duty of providing that the new out-patients' department shall not be proceeded with, nor shall its position on the site be finally determined until this perfected plan has been prepared with the aid of all the expert and medical opinion available. When it has received the formal approval and endorsement of the Governors, as well as that of the active medical staff, the former will be in a position to issue an appeal which should prove successful.

### "Unemployed" Processions.

The report of a "Home of Shelter" in the East End casts a depressing light upon the means by which the out-of-work bring their woes before the public. The compiler of that report declares that the processions of unemployed make the establishment of genuine relief works difficult. It is easy to believe this statement. These processions are a form of medicancy, tolerated by custom and encouraged by the cheap sympathy of those who buy comfort of conscience for a copper; but as consciences of that kind soon get satiated, the takings of the processionists soon fall, and they are no nearer to steady work than before, while they are rather demoralised by such casual alms as may have come to them. It is to be feared that the winter upon which we have entered will be a hard one for many. The uncertainty which prevails in the world of business will affect all, from the highest to the humblest. So we may soon look for the inevitable marching of the unemployed. It is possible that the display of hunger and misery may have a very good effect, if it drives those who see it to inquire into the methods of helping the distressed in their neighbourhood or in the poorer parts of the city, and to contribute to them according to their means. But if it only tempts them to give casual doles to the processionists, the money will be spent, at best, in mere temporary relief, and may possibly become absolutely demoralising.

### The Coroner's Jury.

There are many respects in which the coroner's inquest is open to criticism, and it is highly probable that sooner or later a determined effort will be made radically to reform its methods and procedure. Among the various suggestions advanced by the advocates of a new departure is a proposal that the coroner shall sit without a jury and shall be advised on technical points by properly authorised assessors. This position has recently been discussed by Mr. Troutbeck in a paper read by him before the newly constituted Medico-Legal Society. It is noteworthy that Mr. Troutbeck is strongly in favour of the retention of the jury system. As a coroner of large experience who must at various times have suffered irritation and annoyance from the eccentricities of individual jurymen, it would not have been altogether surprising had he adopted a different attitude. Other reasons apart, the jury system has this important claim, that it secures the confidence of the public. A verdict or decision attested by legal or other experts may be capable of a more logical defence, but, without question, the finding of a jury carries a degree of weight to the general mind that the more scientific opinion fails to command. Indeed it is hardly too much to say that there is much popular distrust of officialism which applies to judicial proceedings at least as much as to general affairs. The expert may give his opinion and advice, but there is a widespread feeling that the final decision must be left to the judgment of common sense. This principle is being acted on to an increasing extent in public life, and thus the jury system may claim the sanction of modern tendencies not less than that of tradition and usage. All this may fairly be urged in favour of the coroner's jury provided the method of selection is conducted in a proper manner. If the list of voters is impartially and rigidly followed there can be no doubt that in the enormous proportion of instances the jury so selected will represent a fair average of intelligence and judgment. But a slipshod practice which leaves the constitution of a jury to be settled by bargain with the coroner's officer is both bad in itself and prejudicial to the entire jury system.

